# Research on Object Detection of PCB Assembly Scene Based on Effective Receptive Field Anchor Allocation

**DOI:** 10.1155/2022/7536711

**Published:** 2022-02-14

**Authors:** Jing Li, Weiye Li, Yingqian Chen, Jinan Gu

**Affiliations:** ^1^School of Mechanical Engineering, Jiangsu University, Zhenjiang 212000, China; ^2^School of Electronic Information and Electrical Engineering, Anyang Institute of Technology, Anyang 455000, China; ^3^School of Mechanical and Electrical Engineering, Guangdong University of Technology, Guangzhou 510006, China

## Abstract

Vision-based object detection of PCB (printed circuit board) assembly scenes is essential in accelerating the intelligent production of electronic products. In particular, it is necessary to improve the detection accuracy as much as possible to ensure the quality of assembly products. However, the lack of object detection datasets in PCB assembly scenes is the key to restricting intellectual PCB assembly research development. As an excellent representative of the one-stage object detection model, YOLOv3 (you only look once version 3) mainly relies on placing predefined anchors on the three feature pyramid layers and realizes recognition and positioning using regression. However, the number of anchors distributed in each grid cell of different scale feature layers is usually the same. The ERF (effective receptive field) corresponding to the grid cell at different locations varies. The contradiction between the uniform distribution of fixed-size anchors and the ERF size range in different feature layers will reduce the effectiveness of object detection. Few people use ERF as a standard for assigning anchors to improve detection accuracy. To address this issue, firstly, we constructed a PCB assembly scene object detection dataset, which includes 21 classes of detection objects in three scenes before assembly, during assembly, and after assembly. Secondly, we performed a refined ERF analysis on each grid of the three output layers of YOLOv3, determined the ERF range of each layer, and proposed an anchor allocation rule based on the ERF. Finally, for the small and difficult-to-detect TH (through-holes), we increased the context information and designed improved-ASPP (Atrous spatial pyramid pooling) and channel attention joint module. Through a series of experiments on the object detection dataset of the PCB assembly scene, we found that under the framework of YOLOv3, anchor allocation based on ERF can increase mAP (mean average precision) from 79.32% to 89.86%. At the same time, our proposed method is superior to Faster R-CNN (region convolution neural network), SSD (single shot multibox detector), and YOLOv4 (you only look once version 4) in the balance of high detection accuracy and low computational complexity.

## 1. Introduction

With the increasing influence of electronic products in social changes, major countries regard electronic product manufacturing as a strategic development industry. Completing the intelligent production transformation and upgrading the electronic product manufacturing industry is also an inevitable choice for the manufacturing industries of all countries. In particular, the realization of visual object detection in the entire manufacturing field of electronic products can help manufacturers eliminate labor shortages and improve product competitiveness.

At present, visual object detection has been widely used in different stages of electronic product manufacturing, such as the manufacture of electronic components, PCB surface mounting, and reliability testing. However, the THT (through-hole technology) in PCB assembly requires highly skilled operators trained in the corresponding standards to complete it. These manual placement costs are high and become the bottlenecks of the intelligent manufacturing of electronic products. The most challenging problem in the THT process of PCB assembly is the messy placement of electronic components and the extremely small vias. Even a well-trained operator uses vision to recognize these electronic components with different shapes and directions and similar-looking vias with a high error rate. Therefore, studying the problem of intelligent visual object detection involving THT in PCB assembly scenarios can help electronic product manufacturers increase output, improve quality, and significantly reduce costs.

The task of object detection [1–4] is to find all the objects of interest in the image and determine their position and class. Deep convolutional neural networks (DCNN) [5–8] is a biologically inspired structure for hierarchical computing features. After the concept of DCNN was presented, object detection has achieved remarkable progress based on deep learning techniques [9]. For a long time, improving the detection speed and accuracy has been the mainstream problem in the research field of vision-based object detection algorithms. Anchor box is the key to improving the quality and speed of object detection using DCNN. Since the introduction of the RPN (region proposal network) proposed by Faster R-CNN [10], the idea of selecting a bounding box based on an anchor has been widely used. An anchor is a set of fixed reference frames of different scales and positions according to the size of varying label samples in the training data set. These reference frames cover almost all positions in the selected feature pyramid layer. Each reference frame is responsible for comparing with ground truth to obtain regional proposals larger than the IoU (intersection over union) threshold. These proposals are retained as bounding boxes using NMS (nonmaximum suppression). The bounding box contains the location and class information of the detection object. At present, most of the mainstream object detection methods use the anchor, and the experiment proves that the effect is significantly improved compared with the previous sliding window.

As a typical representative of the one-stage object detection algorithm, the YOLO series of algorithms have attracted much attention from the day of its birth because it can realize multiobject detection in real-time [11]. In the development process from YOLOv1 to YOLOv5 [12], we always use multiple anchors as dense detectors to improve the accuracy and speed of object detection. As we know, the YOLOv1 network structure is simple, with only one output layer and two anchors; however, each grid of the output layer can only predict one class. For YOLOv2 [13], after the input image was preprocessed and resized to 416 × 416, the backbone network of YOLOv2 made the image pass through the deep convolution became 13 × 13. At this layer, five anchors of different sizes are predetermined from the training data and are fed to the model as a preselection box before training and prediction. The increase of anchors makes training faster by determining the shape and size of possible bounding boxes. The main problem with YOLOv2 is that it cannot adapt to more pictures and only detects one layer. Unlike YOLOv1 and YOLOv2, which predict the output at the last layer, YOLOv3 [14], YOLOv4 [15], and YOLOv5 are all nine anchors generated by clustering training data, and these nine anchors are equally distributed to three output layers of different scales according to the size to achieve object detection.

The 3-3-3 of the fixed allocation of the anchors method of evenly distributing anchors on three output layers of different scales makes full use of the idea of feature pyramid and dense box search and achieves excellent accuracy. However, this anchor allocation mechanism does not consider that the anchor size generated by clustering is random. The ERF size of each grid of the three anchor allocation layers is fixed. In DCNN, the ERF reflects the size of the effective area when each pixel of each feature layer is mapped to the input image during the convolution process [16]. We can imagine that if the anchor size assigned to the grid is much larger than the ERF size of the grid, then the extracted feature maps are only a tiny part of the object to be detected. The entire network detection object seems like a blind person touching the elephant. The anchor size to the grid is much smaller than the ERF size, and hence, the extracted feature map contains too much context information that it is easy to cause interference. The entire network detection object is like finding a needle in a haystack. However, few people associate ERF with the effectiveness of the anchor to improve the accuracy of target detection.

To solve the above problems, we choose YOLOv3 as the object detection framework, take the THT electronic components and through-holes in the PCB assembly scene as the detection objects, take the ERF size range of the anchor distribution layer as the entry point, and design the ERF-based anchor allocation rules to improve detection accuracy. The main contributions of this paper are summarized as follows:

1. We proposed a new dataset for object detection in PCB assembly scenes. This dataset involves three THT scenarios: before assembly, during assembly, after assembly, and 21 classes of detection objects.
2. We have realized the refined analysis of the three output layers of YOLOv3 from the perspective of ERF and proposed an ERF-based anchor allocation rule.
3. We designed an improved-ASPP and channel-attention joint module for TH with a small size and similar appearance. These modules increase the context information, adjust the weights of different channels in the feature layer, and improve detection accuracy.

The following section reviews object detection methods in manufacturing scenes and various improvement methods based on anchors. In section 3, we describe the proposed method. In section 4, we present experimental results and show the effectiveness of the proposed method. Finally, section 5 concludes this paper.

## 2. Related Work

As the ERF-based anchor allocation method for PCB assembly scene object detection involves object detection in manufacturing scenes and anchor-based improvements, the following will introduce the related work in these fields.

### 2.1. Object Detection in Manufacturing Scenes

With the development of the manufacturing industry, intelligent manufacturing technology has gradually become the key technology to realize the knowledge, automation, and flexibility of manufacturing to achieve rapid response to the market [17–19]. In particular, vision-based object detection to detect objects in manufacturing scenes can effectively improve intelligent production. There have been some research results on object detection in manufacturing production scenarios in the early stage. Ghadai et al. proposed using 3D-CNN to identify difficult-to-manufacture drilled holes in CAD geometry. This detection can assist in the realization of intelligent manufacturability decisions [20]. Lin et al. proposed a YOLO-based capacitance detection method for PCB assembly, however, the detection object only contains nine types of capacitance [21]. Tao Xian et al. proposed a CNN detection model for five defects likely to occur in the spring wire sockets during manufacturing. This model performs defect detection and classification tasks simultaneously to improve detection efficiency [22]. Lemos et al. proposed using transfer learning, SSD300, SSD512, and Faster R-CNN to achieve efficient detection based on the task of additive manufacturing target recognition [23]. Li et al. studied the role of CAD synthetic data in the semantic segmentation algorithm of industrial manufacturing [24]. For the four small parts of the watch assembly process, Qian et al. proposed IoMA-NMS and a new loss function based on NMS based on the excessive number of preselected frames during the segmentation process, improving the detection effect of Mask R-CNN [25]. At the industrial manufacturing site, Park et al. used wearable augmented reality smart glasses to render the scene through the three-dimensional spatial information of the scene target extracted by Mask R-CNN to help the operator better recognize and understand the operating object in the actual physical scene [26]. Because of the rapid update of clothing styles in clothing manufacturing companies, it is a complex problem to use deep learning to detect defects on untrained objects on clothing. San-Payo et al. proposed an incremental learning algorithm to solve new target feature learning [27]. Tsai and Chou proposed four CNN-based models for precise positioning in PCB manufacturing and production to achieve exact position and angle detection [28]. Zhang et al. proposed a deep multimodel cascade method that combines single-frame image and multiframe image processing to detect and identify foreign particles for the qualitative detection of liquid pharmaceutical products [29]. Zhang et al. proposed a method that combines Faster R-CNN and R-FCN multichannel features to detect surface defects in the production process of solar panels [30]. Li et al. proposed a lightweight model design method based on ERF and anchor matching to solve the detection problem of electronic components on the assembled PCB [31]. Wang et al. took the worker's action recognition and parts detection at the toy assembly site as the research content. They proposed using Faster R-CNN as the detection method to realize the skill transfer in intelligent manufacturing [32]. Faced with the problem of semantic segmentation of safety vents welded on the battery cover during the production process of power batteries, Zhu et al. proposed a lightweight multiscale attention model to effectively improve product quality detection [33].

### 2.2. Anchor-Based Object Detection

Using anchors as preselected boxes to search and match intensively on images to improve the accuracy and speed of object detection is currently the mainstream method for object detection. At present, many scholars are studying the design and improvement of the anchor box. Zhong et al. proposed a method of adaptively learning the anchor box size according to network capabilities and data distribution. This method solves the problem of fixed anchor box size during training and can improve detection accuracy [34]. When Zhang et al. used Faster-RCNN to detect surgical tools in laparoscopic videos, they followed the anchor point center and feature alignment principle. They proposed a subnetwork for predicting anchor size and reducing the number of dense anchors [35]. Yu et al. designed orientation-guided anchors when detecting rotating objects in remote sensing images. One of the two subnetworks is responsible for training and generating anchors of appropriate size in the anchor generation stage. The other is responsible for counting the occurrence probability of the object, avoiding invalid anchors appearing in the background [36]. Tian et al. used the attention mechanism to produce adaptive anchors to improve object detection in remote sensing images [37]. In the task of license plate detection, Nguyen et al. designed the anchors of fixed length and proportion at different layers of the feature pyramid according to the aspect ratio specified by the license plate. They used a multiscale regional proposal network with predicted anchor position information to finally improve the detection effect [38]. Ma et al. considered the spatial relationship between anchor, ground truth, and bounding box for a one-stage anchor-based object detection algorithm. They proposed a location-aware framework to improve the detection effect and robustness [39]. Li et al. proposed posture anchors when using a one-stage object detection framework for hand keypoint detection. Through the clustering analysis of multiple gestures, angles, and scales of representative posture anchors, the occlusion problem of hand key point detection can be solved [40]. In the object detection task, Jin et al. proposed to use the ground truth area in the train set to adaptively generate anchors for different feature layers in the backbone to reduce the number of anchors [41]. Hosoya et al. found that in video object detection, there will be instantaneous object loss in consecutive frames. They proposed to use a soft threshold to solve the problem of fixed-value anchor and threshold for the same object scale and angle change in consecutive frames [42]. Given the multiscale and denseness of remote sensing image targets, Guo et al. adopted a nonfixed k-value clustering algorithm, used the principle of minimum clustering loss, and generated the number of adaptive anchors according to the characteristics of the object data in the training set [43]. Grabel et al. used circular anchors to replace the rectangular anchors commonly used in object detection when detecting the blood cell [44]. Chen et al. proposed generating K-means anchor boxes based on similar shape distances aiming at the problem of small size and a large number of ship detection in synthetic aperture radar images [45]. Zhu et al. used the size ratio relationship between the feature layers to allocate the anchors obtained by the K-means according to the same ratio, which improved the object detection effect of multiscale remote sensing images [46]. When detecting ships in complex backgrounds, Xiao et al. proposed using pairwise semantics in the direction and horizontal minimum bounding boxes, resulting in fewer and more accurate rotation anchors, reducing the complexity of calculations [47]. Wang et al. proposed a receptive field generation method that matches the anchor size. They used a nonsquare convolution kernel to generate the receptive field closest to the anchor size and redistribute the size of the anchor using the relationship between the vehicle space position [48]. When performing object detection, Wang et al. considered invalid anchors to affect the detection speed and proposed an anchor position prediction network and an anchor shape prediction network, which greatly reduced the number of anchors and computation time [49]. In the multiclass object detection problem of remote sensing images, Mo et al. proposed a class-specific anchor generation algorithm to improve the recall rate and designed the most suitable anchor for each class [50]. Gao et al. found that the same object has the same aspect ratio even though the obtained field of view is different and proposed an anchor that only predicts the aspect ratio, which improves object detection accuracy [51]. In the task of pedestrian detection, Fang et al. found that geometric constraints of pedestrians can be combined with anchor frame information, and anchors containing geometric constraints are used to reduce reasoning time and reduce the error rate of reasoning in pedestrian detection [52]. Deng et al. used the learned proposals in the two-stage R-CNN to propose a learnable anchor and replaced the learnable anchor with the fixed anchor in the one-stage to achieve real-time scene text detection [53]. In the object detection task of optical remote sensing images, BAO et al. proposed two regressions of anchors that adapt to different IOU thresholds according to the different needs of recognition and positioning [54]. Zhang et al. proposed an optimized sampling anchor constructed using feature maps extracted from deep learning. This method is suitable for two-stage object detection to improve performance [55]. Yang et al. proposed a meta-anchor and designed an anchor function to learn parameters from preselected boxes. The setting of this anchor and the distribution of bounding boxes are more robust in object detection tasks [56].

### 2.3. Knowledge Gaps

Although significant progress has been made in the two fields mentioned above, some gaps still need to be fulfilled from the review.

For object detection tasks in manufacturing scenarios, many studies have involved different processes of manufacturing, such as drawing geometry detection in production design, tool material detection in the early stage of production, action recognition in the production process, and defect detection in production products. Especially defect detection in the manufacturing process is a research hotspot. However, these object detection tasks involve a single production scene, each scene has fewer object classes, and the object distribution is relatively scattered. The PCB assembly scenarios proposed in this paper include three different situations, the scattered electronic components before assembly—some electronic components are inserted into the TH on the PCB during the assembly—and all the inserted TH on the PCB after assembly. There are 21 classes. The object to be detected has a significant difference in scale and a high similarity in appearance. As far as we know, this is the first full-scene detection dataset for electronic component assembly.

As far as anchor-based object detection methods are concerned, the main research points are adaptive anchors that can be trained and learned, anchor alignment, arbitrary-oriented anchor design, and anchor effectiveness research. Most of the experts only pay attention to the anchor itself, ignoring the relationship between the corresponding size of the anchor and the ERF size corresponding to each grid of the anchor placement layer when the anchor performs the function of the preselection box. This paper proposes an anchor allocation algorithm that only needs to use the basic K-means algorithm to generate anchors and allocates based on the principle that the anchor size placed on each grid on the output channel matches the ERF size corresponding to each grid. As far as we know, this is the first time that the ERF size of each grid in the output feature layer of the object detection model has been quantified, which provides a basis for the effectiveness of the allocation of anchors and improves the performance of object detection.

## 3. Methodologies

**Figure 1:**
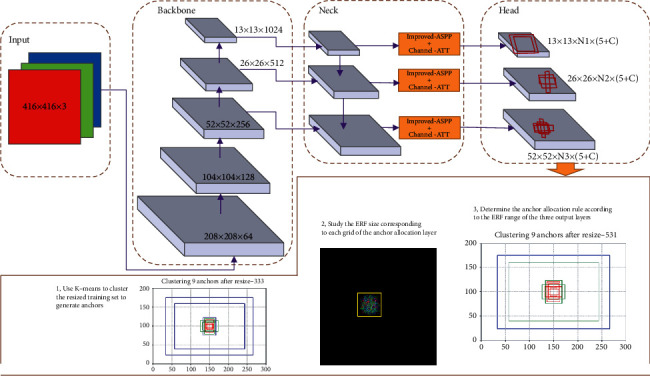
Description of anchor allocation method based on ERF. Object detection based on YOLOv3 is achieved by placing dense anchors on the three output channels of the head through regression to achieve object recognition and positioning. The red rectangular boxes in the head part represent anchor boxes placed in different output layers. N1, N2, and N3 represent the number of anchors allocated to the three output layers. The allocation of the original anchor is 3-3-3. Blue, green, and red represent the anchors placed on each grid on the 13 × 13, 26 × 26, and 52 × 52 layers. The yellow rectangle represents the ERF size corresponding to a grid. Grids with different positions correspond to different ERF sizes. According to the ERF size range of the anchor placement layer, redistributing the number of anchors will improve the detection effect of the original anchor even distribution.

This study uses anchor-based one-stage object detection network YOLOv3 as the primary network. The electronic components, PCB, NITH (no-inserted through-holes), and ITH (Inserted through-holes) are detection objects in the electronic component assembly scene. Analyzing and calculating the ERF of each grid is the starting point of this research. This research focuses on determining the anchor distribution principle according to the ERF size range of each anchor distribution layer.  Figure 1 shows the entire anchor allocation method based on ERF. As we know, an object detection network structure based on CNN consists of four parts: input, backbone, neck, and head. For YOLOv3, the input is a 3-channel color image resized to 416 × 416. The backbone is a feature extractor in the form of a feature pyramid. Here, we choose Darknet-53 as the backbone. The neck is a feature fusion model that helps the detector to locate and recognize objects better. The head has three convolution outlets, and each outlet completes the object detection of different scales using the assigned anchor. The method we propose includes ERF-based anchor assignment and a joint feature fusion enhancement module adapted to difficult-to-detect targets, which occur in the head and neck of YOLOv3, respectively, which will be described in detail below.

### 3.1. Anchor K-Means

K-means is used in the anchor generation problem of object detection as a simple and commonly unsupervised learning algorithm. K groups of anchors with high similarity in width and height are automatically generated by clustering the bounding boxes of the training set.

We choose 416 × 416 as the size of the YOLOv3 input image. All training set and test set images will be resized to 416 × 416 first, and the resized bounding box size is used for clustering to generate anchors. An anchor is used as a preselection box for object detection. We can obtain the empirical size of the object from a large amount of label data in the training set and quickly find the detected object using regression training CNN. Why should it be emphasized that the anchor we used was generated after the original image size was resized to 416 × 416? There are two reasons. One is that for YOLOv3, whose input image size is fixed at 416 × 416, it is more accurate to normalize the anchor to 416 for object detection. The other is the anchor size can directly compare with the ERF size to determine the anchor distribution rules.

**Figure 2:**
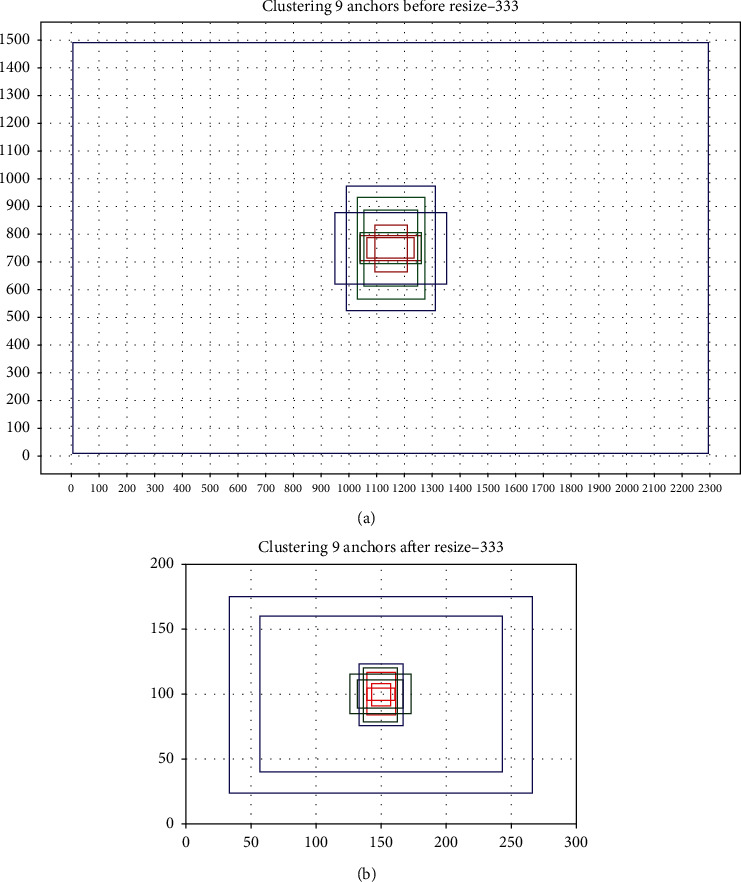
Use K-means to generate nine anchors. The blue, green, and red represent the anchors placed on each grid on the 13 × 13, 26 × 26, and 52 × 52 layers. (a) Generate nine anchors before resize. (b) Generate nine anchors after resize.

Here, we compare the anchor generated by clustering the PCB assembly scene training dataset before and after resizing to 416. As a professional camera is used to obtain pictures of the assembly scene, the photo size is large, and it is 4092 × 3000. The objects to be detected include PCB, capacitors, inductors, chips, ITH, NITH, etc. The size difference between these objects is large. As we all know, an object can only have one anchor responsible for detection. To avoid the missed detection of stacked objects, YOLOv3 sets three anchors on each grid cell of the three outlets, a total of 9 anchors. We follow this idea of setting the number of anchors using the nine anchors generated after resizing as the anchors to be allocated. The anchor size obtained by clustering before resizing is [171, 72], [218, 87], [118, 168], [220, 110], [194, 274], [242, 364], [400, 255], [320, 448], and [2293, 1480], as shown in Figure 2(a). After resizing all images, perform K-means clustering to generate anchor sizes. They are [9, 21], [14, 17], [22, 32], [22, 35], [27, 41], [30, 47], [34, 47], [186, 120], and [233, 151], as shown in Figure 2(b).

### 3.2. Refined Analysis of the ERF Size

**Figure 3:**
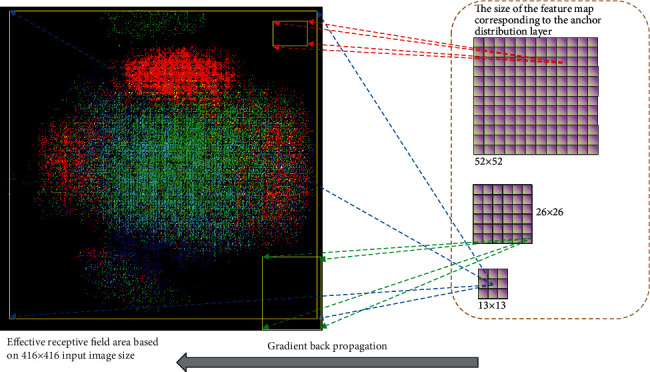
Schematic diagram of refined analysis of ERFs. The entire analysis and calculation are based on gradient backpropagation. The colored dots on the black background are the effective areas that can be activated when the grid in the seventh row and the seventh column on the 13 × 13 feature map is mapped back to the original image. The yellow rectangle is the smallest enclosing rectangle of the ERF we get. The three rectangular boxes, respectively, represent the size and position of the ERF corresponding to a grid in the three output layers. The three grids on the right represent the size of the three output layers when the input image size is 416 × 416. These three output layers are also anchor distribution placement layers.

In the deep neural network, a concept called the receptive field represents the size of the range of perception of the original image by neurons in different positions within the network. The deeper the number of convolutional layers, the larger the field of view that the end pixel will be fed back to the original image. The ERF refers to the area of the original image information that can be effectively received, and this area has a Gaussian distribution. When the convolutional neural network uses the convolution kernel to perform traversal feature extraction on the image, pixels at different positions in the previous layer contribute differently to the feature value of a point in the current layer [57]. Other researchers have researched the calculation and analysis of the ERF of convolutional neural networks [31, 57–59]. Different researchers have studied the accounting and analysis of the ERF of convolutional neural networks. However, current pieces of research default to the fact that ERF is square, and there is no detailed analysis of their size and shape. We use the gradient backpropagation method shown in Figure 3 to perform ERF analysis and calculation on the three output layers of YOLOv3, i.e., each grid corresponding to the allocation anchor.

The entire method of refined analysis of ERFs is divided into seven steps.

  Step 1. Load the object detection model. As the object detection framework used in this study is YOLOv3, it is necessary, firstly, to construct and load this model.
  Step 2. Import model weights. Use the weights of the pretrained YOLOv3 object detection model to assign values to the grid of the output layer, perform gradient backpropagation, and determine the activation area.
  Step 3. Determine the number of feature layers to be analyzed. The ERF analysis method proposed in this paper can be used to analyze any pixel in any feature map corresponding to the original image. Therefore, we must first inform the layer number of the feature map to be studied in the entire object detection model.
  Step 4. Assign the initial value of 1 to each pixel of the feature layer to be analyzed. The essence of refined analysis lies in precision and refinement. Determining the size of the original image area that the feature map point can feel by point will play a particular role in the visual task of the convolutional neural network.
  Step 5. Use gradient backpropagation to calculate the activation degree of the three channels of the input image. Firstly, construct three 416 × 416 black channels, representing the original input color image's *R*, G, and B channels. In turn, the feature map with the brightness of each pixel point of 1 is backpropagated back to the *R*, G, and B channels of the original image to obtain a three-channel activation map.
  Step 6. Use the Gaussian function to determine the ERF. As far as we know, not all the activation points obtained in step 5 are valid, and the effective area is Gaussian. Using the two-sigma rule of Gaussian distribution, it can determine the ERF area where each pixel on the feature map to be detected is mapped back to the original image.
  Step 7. Determine the ERF size. After clarifying the ERF range, determine the ERF size according to the leftmost, rightmost, bottom, and top positions of the activation points distributed in the range of 416 × 416.

Among the above steps, the sixth step is the most important. Using Gaussian distribution to determine the ERF range is the basis of this research.

### 3.3. Anchor Allocation Rules

With a refined analysis method for the ERF of each pixel of the feature map, the ERF range of the entire feature map is determined. The YOLOv3 object detection network relies on placing anchors on each grid of the three output layers of the head to finally achieve object detection using regression. Once the object detection task's training set is selected, the corresponding anchor's size is determined. Compared with the ERF size of the detection layer where the anchor is placed, the size of the anchor is too small, like finding a needle in a haystack, and too big, like a blind person touching an elephant.

**Figure 4:**
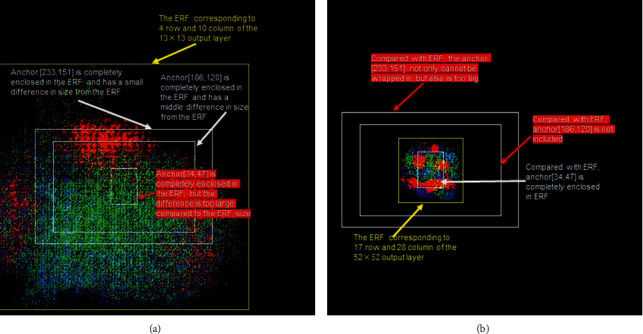
A schematic diagram of a grid corresponding to the ERF and assigning three anchors. The yellow rectangle indicates the range of the ERF. The three white rectangular boxes represent the three anchors allocated by this output layer. (a) 13 × 13 output layer, the 4^th^ row and the 10^th^ column grid ERF and anchor. (b) 52 × 52 output layer, the 17^th^ row and the 28^th^ column grid ERF and anchor.

How to determine the rules for assigning anchors based on the ERF? Here, we use Figure 4 to illustrate what kind of anchor allocation is appropriate and what kind of anchor is inappropriate. From Figure 4(a), we can see that although the three anchors are all enclosed in the ERF because the sizes of the anchors are different, we find that the anchor [34, 47] is too small compared to the ERF. An anchor in this output layer is like finding a needle in a haystack for object detection, which is inappropriate. From figure (b), we can see that only one anchor is enclosed in the ERF, and the other two anchors [186, 120] and [233, 151] are larger than the ERF. The boundaries of these two anchors are both outsides of the ERF. When such two anchors are placed in this layer for object detection, it is like a blind person touching an elephant. Each time, only a part of the object can be detected, which is inappropriate.

The above analysis shows that the first condition for assigning a suitable anchor is that the anchor should be enclosed in the ERF, and the difference between the anchor and the ERF should not be too large. Therefore, we design an anchor allocation rule based on ERF. According to the ERF of each grid of the anchor placement layer, the ERF range of the entire output layer can be obtained. The anchors can be arranged from small to large. The larger the feature map of the output layer, the smaller the ERF size corresponding to each grid. The smaller the feature map of the output layer, the larger the ERF size corresponding to each grid. We start with the smallest anchor and compare it with the ERF corresponding to all grids in the largest feature map of the output layer. Suppose this anchor can be enclosed in all ERF. In that case, this anchor should be allocated in this layer, as long as one ERF cannot contain it, and this anchor will enter the second largest feature map of the output layer for comparison and allocation. Repeat afterward until all anchors are allocated to different layers.  Algorithm 1 shows the pseudocode corresponding to the anchor enclosed algorithm.

Through the above anchor allocation algorithm based on ERF, we changed the original YOLOv3 anchor average distribution algorithm. We reallocated the anchors in the three output layers, respectively, and the anchors of each layer can be enclosed corresponding to all grids in this layer. At the same time, follow the principle that the anchors of different layers are no longer reused because the ERF size corresponding to the 52 × 52 output layer is the smallest. The anchors allocated to 52 × 52 are those that the ERF of this layer completely encloses. For the 26 × 26 output layer, the anchors that can be assigned are those that are completely enclosed by this layer after removing the anchors that have been allocated by 52 × 52. For the 13 × 13 output layer, firstly, remove the anchors from all anchors where 26 × 26 and 52 × 52 have been used, and the remaining anchors are allocated to this layer. Finally, an anchor allocation algorithm based on the ERF is realized.

### 3.4. Some Other Improvement

**Figure 5:**
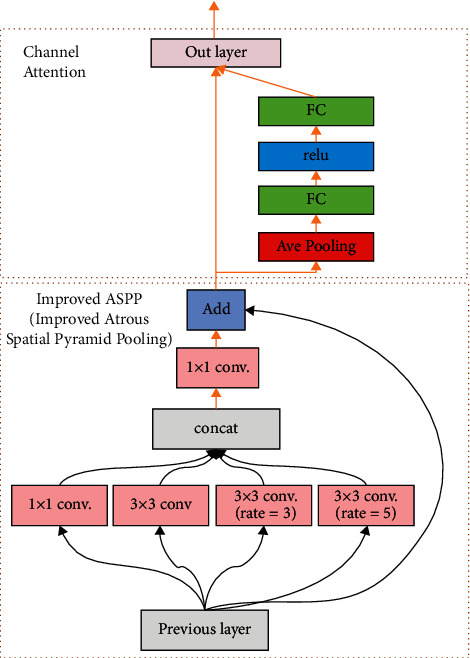
A schematic diagram of the improved-ASPP and channel attention combined module. The rate stands for dilation rate. This joint module will be used to connect the neck and head of the original YOLOv3. It is used three times for the three output channels, however, the input and output layers are adjusted according to the number of feature channels extracted by the neck part. The output of the entire joint module will be input to the corresponding head layer.

In addition to assigning suitable preselected boxes to object detection, to improve the detection effect further, we also combine context and channel attention to improve multiscale feature fusion. For the neck part of YOLOv3, the feature maps extracted by deep learning, we let them pass through an improved-ASPP [60] and channel attention joint module and send them to the final head part.  Figure 5 shows the entire joint module.

Spatial pyramid pooling with atrous/dilated convolution (ASPP) can simultaneously process objects of different sizes. It has been applied in the field of semantic segmentation and object detection. The improved-ASPP proposed in this paper concatenates contextual multiscale feature information on the original feature map. Then, the concatenated result and the original feature map are added together. Whether concatenation or add, it integrates feature information of different scales. The improved-ASPP strengthens the reuse of features and solves the problem of gradient disappearance in the deep network.

As everyone knows, the attention mechanism can ignore irrelevant information through the model and focus on the critical information we want it to focus on. The channel attention is to improve the network's representation ability by modeling the dependence of each channel, especially to learn the weight of different channel features in object detection, and to adjust the features channel by channel, so that the network can learn to use global information to selectively enhance features that contain helpful information and suppress useless features. Here, we connect the channel attention module after the improved-ASPP module to improve the detection performance further.

## 4. Experiments

We validated the proposed method on the PCB assembly dataset. Therefore, in the experimental part of this section, we will, firstly, introduce the constructed PCB assembly scene dataset. Secondly, analyze the ERF of the three different output layer grids based on YOLOv3. Then, use the anchor allocation rules based on the ERF proposed in this paper. The anchors are allocated in different output layers. Finally, the experimental results are analyzed and discussed.

### 4.1. PCB Assembly Dataset

Based on the background of intelligent manufacturing, we designed a PCB assembly scene dataset. The so-called PCB assembly places electronic components in a designated position on the bare PCB. There are currently two main types of PCB assembly technology: through-hole technology (THT) and surface mount technology (SMT). The vast majority of PCB assembly uses a mixture of the two technologies. SMT has achieved automation at present, mainly because THT still needs to rely on manual completion. We are concerned about detecting the electronic components to be inserted and the corresponding TH on PCB after PCB completes SMT.

**Figure 6:**
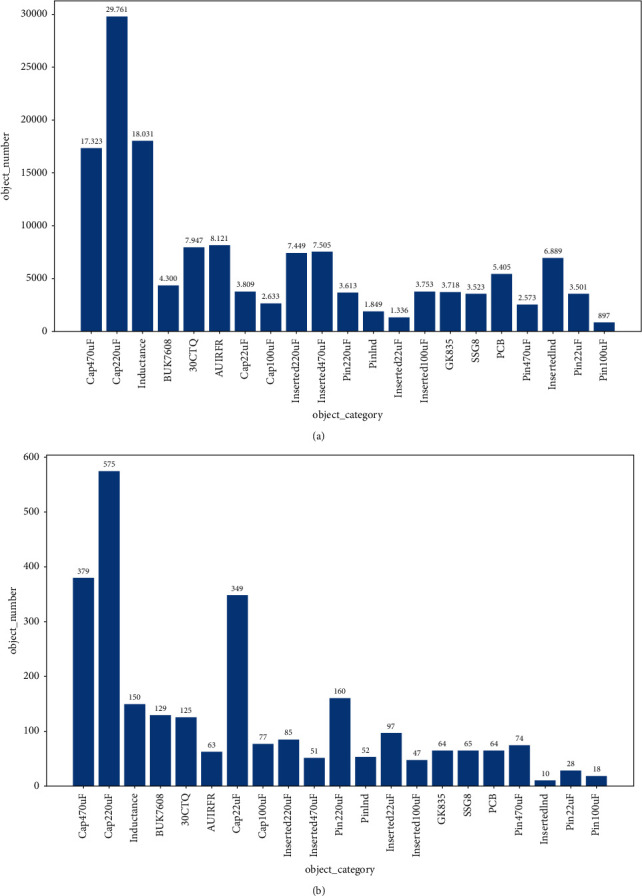
A statistical graph of the number and category of training and test datasets for PCB assembly scenes. The horizontal axis represents the categories in the dataset. The vertical axis represents the number of objects in each class. (a) Number and category of training data. (b) Number and category of test data.

The dataset contains three scenes before assembly, during, and after assembly and 21 class objects. Among these detection objects, there are four types of capacitors with small variance between classes, preinsertion, postinsertion through-hole with slight differences in appearance, and large-size PCB. There are 9636 pictures in the whole dataset. The size of all pictures is 4092 × 3000. The picture background is white. The electronic components outside the PCB are messy and disordered. The electronic components and TH on the PCB are fixed and sequence relative to the PCB. We randomly divide the training data and the test data according to the ratio of 8 : 2 to the entire dataset.  Figure 6 shows the object number and category statistics of the training set and test set of the whole PCB assembly scene.

### 4.2. ERF Refined Analysis of YOLOv3 Anchor Allocation Layer

Performing refined ERF analysis on the three anchor allocation layers of YOLOv3 and determining the ERF range of the three feature layers is the basis for realizing ERF-based anchor allocation. According to the method proposed above, we can calculate the ERF size corresponding to each grid under the three sizes of 52 × 52, 26 × 26, and 13 × 13. To vividly illustrate the different positions and sizes of the ERF corresponding to each grid of each layer, we have designed three tables. The first column of each table indicates the layer's size, and the second column, x##y, represents the *x* row and *y* column in the grid. The third column represents the schematic diagram of the ERF corresponding to the grid. The fourth column represents the size of the ERF corresponding to the grid. We show the ERF of 5 grids randomly selected from 13 × 13, 26 × 26, and 52 × 52 in Tables 1–3, respectively.

Using these three tables, we will find that the larger the output layer, the smaller the ERF corresponding to each grid, and the smaller the output layer, the larger the ERF corresponding to each grid. The ERF size corresponding to each grid is different in the same layer.

### 4.3. Application of ERF-Based Anchor Allocation in PCB Assembly Scenarios

According to the previous analysis of the YOLOv3 model, we learned that there are 169 grids in the 13 × 13 output layer. In terms of area, the smallest ERF size is (222, 162), and the largest ERF size is (390, 399). What we are more concerned about is that the anchors allocated in this layer are entirely enclosed by the effective receptive fields of all grids. Therefore, we find the ERF size with the smallest width (189, 227) and the ERF size with the smallest height (226, 160). In the same way, we can obtain that the ERF size with the smallest area of the 26 × 26 output layer is (84, 111), the ERF size with the largest area is (182, 193), the ERF size with the smallest width is (81, 122), and the ERF size with the smallest height is (112, 86). For the 52 × 52 output layer, the ERF size with the smallest area is (41, 39), the ERF size with the largest area is (118, 87), and the ERF size with the smallest width is (38, 54). The ERF size with the smallest height is (47, 38).

The anchor allocation rules proposed above are applied for the PCB assembly scene training dataset for which the anchor size has been determined. For the 52 × 52 output layer, there are 4 anchors allocated, which are [9, 14, 17, 21, 22, 22, 32, 35]. For the 26 × 26 output layer, there are three allocated anchors, namely [27, 30, 34, 41, 47, 47]. For the 13 × 13 output layer, there are two allocated anchors, [186, 120] and [233, 151]. We have also proposed the improved-ASPP and attention mechanism joint module for YOLOv3. The biggest function of the improved-ASPP is to combine more contextual information and enlarge the receptive field. Therefore, for the joint module improved YOLOv3, we use the same anchor allocation method based on ERF. There are 5 anchors allocated to the 52 × 52 output layer, which are, respectively, [9, 14, 17, 21, 22, 22, 32, 35], and [27, 41]. The 26 × 26 output layer assigns a total of 3 anchors, which are, respectively, [30, 47], [34, 47], and [186, 120]. The 13 × 13 output layer assigns only 1 anchor, i.e., [233, 151].

**Figure 7:**
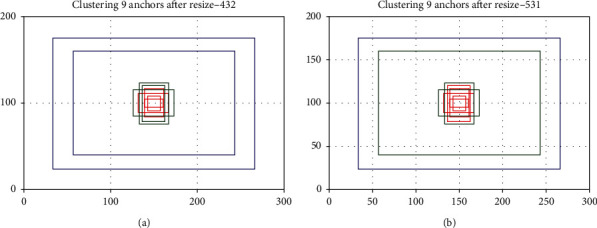
Two anchor allocation schemes are based on ERF. Red, green, and blue represent the anchors allocated by the three output layers of 52 × 52, 26 × 26, and 13 × 13 in the YOLOv3 series model. (a) ERF anchor allocation based on YOLOv3 in PCB assembly scenarios. (b) ERF anchor allocation based on the joint module improved YOLOv3 in PCB assembly scenarios.

Figures 7(a) and 7(b), respectively, show the two anchor allocation schemes obtained by applying anchor allocation rules based on ERF when YOLOv3 and the joint module improved YOLOv3 perform object detection in PCB assembly scenes. It is different from the original YOLOv3, which evenly distributes the nine anchors in the three output layers according to the size.

### 4.4. The Experimental Results

#### 4.4.1. Experimental Platform and Parameter Setting

Experiments were performed on a deep learning workstation with Intel^®^ Xeon^®^ Gold 6132 CPU@2.60 GHz, 192 GB RAM, single NVIDIA^®^ Titan RTX Graphics card 24G, and Ubuntu 18.04 LTS operating system. Program codes were written in Python3.7 [61], using the TensorFlow deep learning library (Version 2.0) [62].  Table 4 shows the parameters used for the experimental algorithms.

To better illustrate the effectiveness of the method proposed in this research, we carried out eight experiments by adding an improved-ASPP, channel attention mechanism, joint module to the neck part, and three anchor allocation schemes.  Table 5 shows the naming and specific improvement steps of these eight sets of algorithms.

#### 4.4.2. Analysis of Subjective Test Results

**Figure 8:**
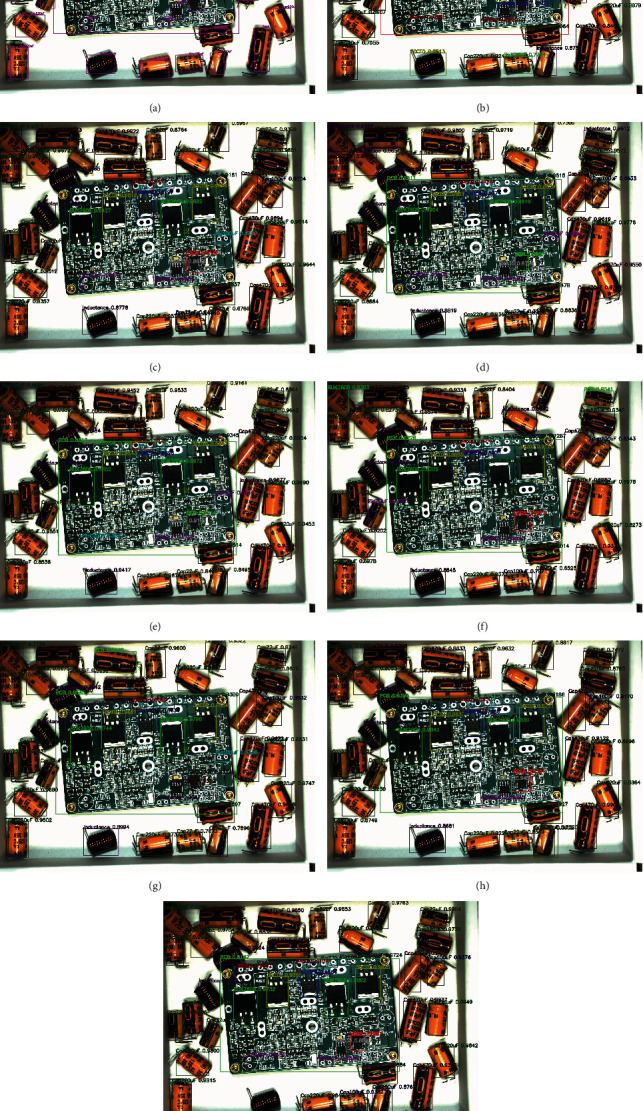
The original image before assembly and the object detection comparison of eight algorithms. (a) Ground truth of original image before assembly. (b) YOLOv3-333+ before assembly. (c) YOLOv3-IASPP-333+ before assembly. (d) YOLOv3-CATT-333+ before assembly. (e) YOLOv3-IASPP-CATT-333+before assembly. (f) YOLOv3-432 +before assembly. (g) YOLOv3-IASPP-CATT-432+before assembly. (h) YOLOv3-CATT-531+ before assembly. (i) YOLOv3-IASPP-CATT-531 (ours)+before assembly.

**Figure 9:**
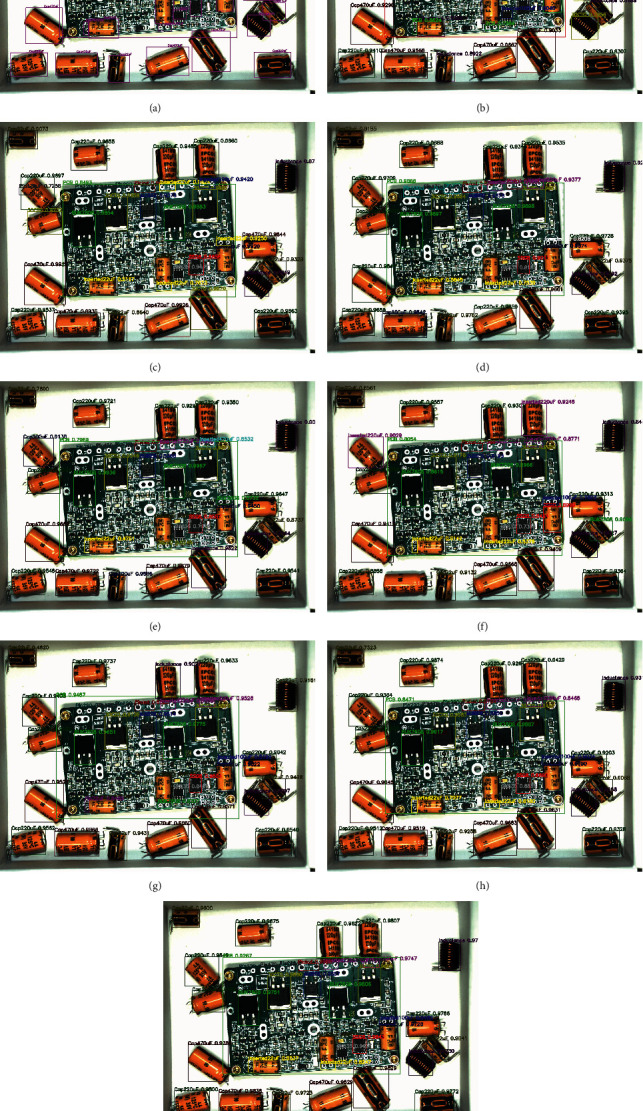
The original image1 during assembly and the object detection comparison of eight algorithms. (a) Ground truth of original image1 during assembly. (b) YOLOv3-333+ image1 during assembly. (c) YOLOv3-IASPP-333+ image1 during assembly. (d) YOLOv3-CATT-333+ image1 during assembly. (e) YOLOv3-IASPP-CATT-333+image1 during assembly. (f) YOLOv3-432+ image1 during assembly. (g) YOLOv3-IASPP-CATT-432+image1 during assembly. (h) YOLOv3-CATT-531+ image1 during assembly. (i) YOLOv3-IASPP-CATT-531 (ours)+ image1 during assembly.

**Figure 10:**
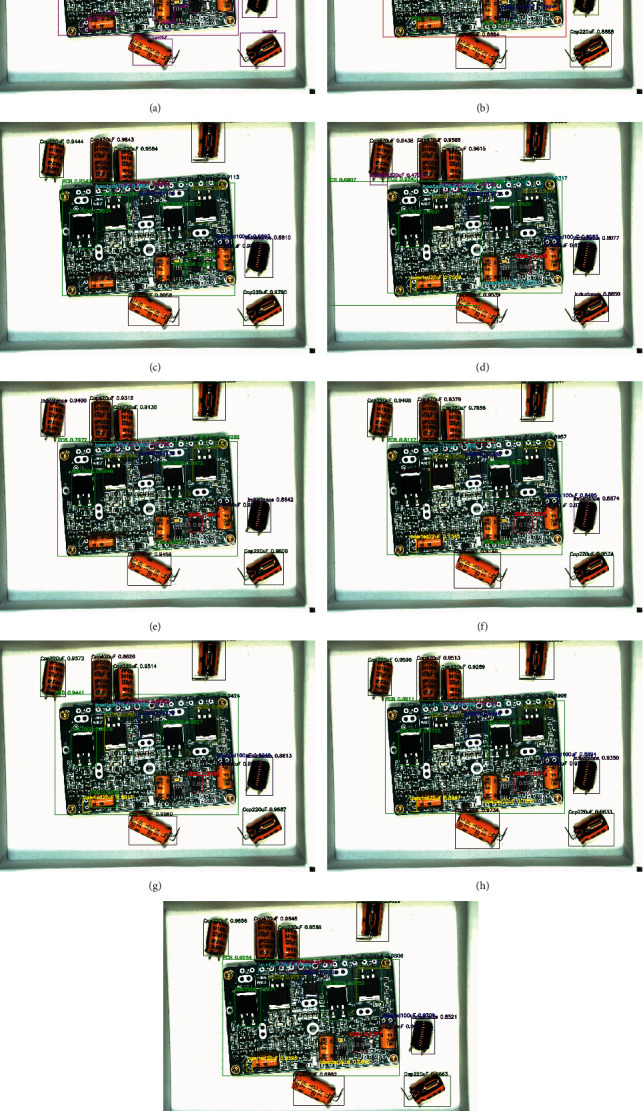
The original image2 during assembly and the object detection comparison of eight algorithms. (a) Ground truth of original image2 during assembly. (b) YOLOv3-333+ image2 during assembly. (c) YOLOv3-IASPP-333+ image2 during assembly. (d) YOLOv3-CATT-333+ image2 during assembly. (e) YOLOv3-IASPP-CATT-333+image2 during assembly. (f) YOLOv3-432+ image2 during assembly. (g) YOLOv3-IASPP-CATT-432+image2 during assembly. (h) YOLOv3-CATT-531+ image2 during assembly. (i) YOLOv3-IASPP-CATT-531 (ours)+ image2 during assembly.

**Figure 11:**
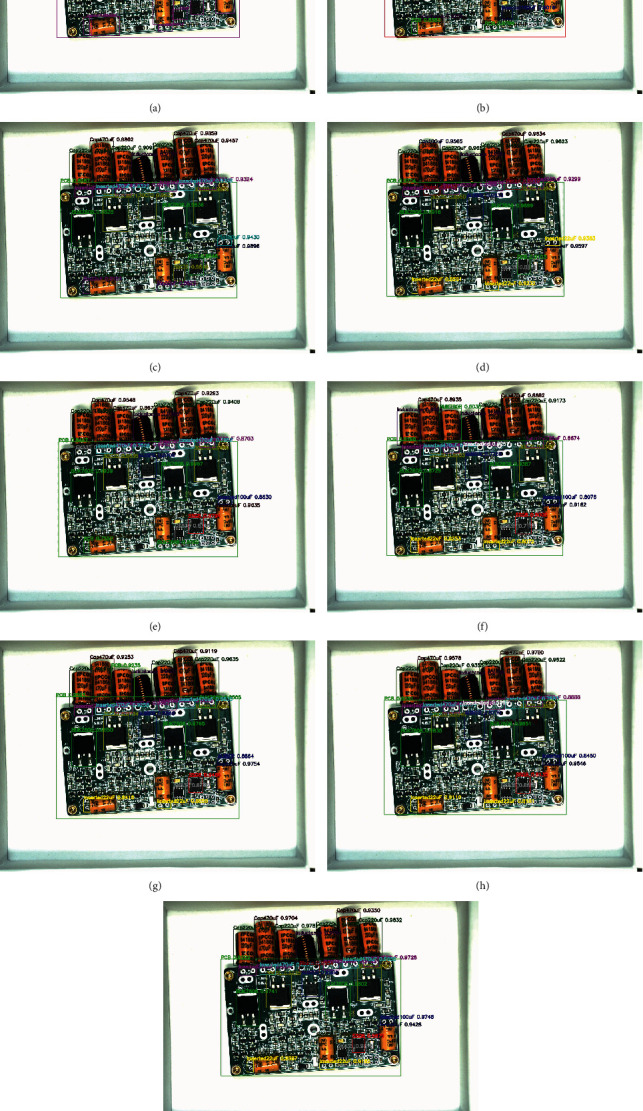
The original image1 after assembly and the object detection comparison of eight algorithms. (a) Ground truth of original image after assembly. (b) YOLOv3-333+ image after assembly. (c) YOLOv3-IASPP-333+ image after assembly. (d) YOLOv3-CATT-333+ image after assembly. (e) YOLOv3-IASPP-CATT-333+image after assembly. (f) YOLOv3-432+ image after assembly. (g) YOLOv3-IASPP-CATT-432+image after assembly. (h) YOLOv3-CATT-531+ image after assembly. (i) YOLOv3-IASPP-CATT-531 (ours)+ image after assembly.

The above eight algorithms can achieve multiobject detection in a PCB assembly scene image, and we tested 120 images with them. Here, we take the detection results of 4 images to illustrate the effect of the eight algorithms. These four images are one before assembly, two during assembly, and one after assembly. In the algorithm test results image, we use rectangular boxes of different colors to indicate the detected object location. The upper left corner of the rectangular box indicates the class and confidence of the object. In Figures 8–11, we, respectively, show the object detection results of four images in three PCB scenarios. Each image contains nine subimages representing the original image's ground truth and the results of the eight algorithms in Table 5.

Upon comprehensively analyzing these four sets of images, we will find that upon only using the average distribution anchors algorithm of YOLOv3 to detect the PCB assembly scene, the error rate is higher, especially on the PCB and large object some chips. With the addition of improved-ASPP and channel attention mechanisms in YOLOv3, we will see the improvement of the detection effect. For example, the detection of PCB gradually approaches the ground truth, and the accuracy of detection of inductance is improved. Especially, when we use the 4-3-2 anchor allocation algorithm based on ERF to detect objects, we will observe that some small objects, such as NITH and ITH, still have low detection accuracy until the improved joint module in YOLOv3 and the anchor allocation of 5-3-1 was performed, which achieved the best detection effect among all algorithms.

### 4.5. Analysis of Objective Test Results

For detecting PCB assembly scene containing 21 classes, we use AP (average precision) to represent the detection accuracy of a single class in the algorithm and mAP (mean of average precision) to represent the average accuracy of all classes in the algorithm. The higher the AP and mAP, the better the detection performance of the algorithm. In Table 6, we use bold to indicate that this algorithm's result is better than or equal to other algorithms. From Table 6, we can learn from the specific data that YOLOv3-IASPP-CATT-531 (ours) improves the detection accuracy in 15 classes, and the mAP obtains the highest value.

#### 4.5.1. Ablation Analysis

For the ERF-based anchor allocation rules, improved ASPP, and channel attention proposed in this paper, we will use detailed ablation analysis in Table 7 to understand the influence of different optimizer functions in the object detection of PCB assembly scenes. We take mAP when the IoU threshold is 0.5 as the measurement standard and use the object detection result of YOLOv3-333 as the benchmark.

A first observation is that the use of ERF-based anchor allocation alone (row 5) brings the most significant increase to mAP (refer to the original YOLOv3-333) in the use of improved-ASPP (row 2), channel attention (row 3), and joint improvement modules (row 4). This result also brings us confidence that the allocation of anchors according to the size range of the ERF of the anchor placement layer can indeed improve the accuracy of detection. As the improved-ASPP can combine more contextual information to expand the ERF size, we considered the joint improvement module of IASPP and CATT in the experiment. We reallocated anchors on the three output layers YOLOv3-IASPP-CATT. A second observation is the joint application of the three modules of improved ASPP, channel attention, and ERF-based anchor allocation (row 8) to obtain the highest value of mAP. Compared with the original YOLOv3-333 (row 1), the mAP of YOLOv3-IASPP-CATT-531 (row 8) has increased by 10.54%.

#### 4.5.2. Analysis of a Series of Curves

**Figure 12:**
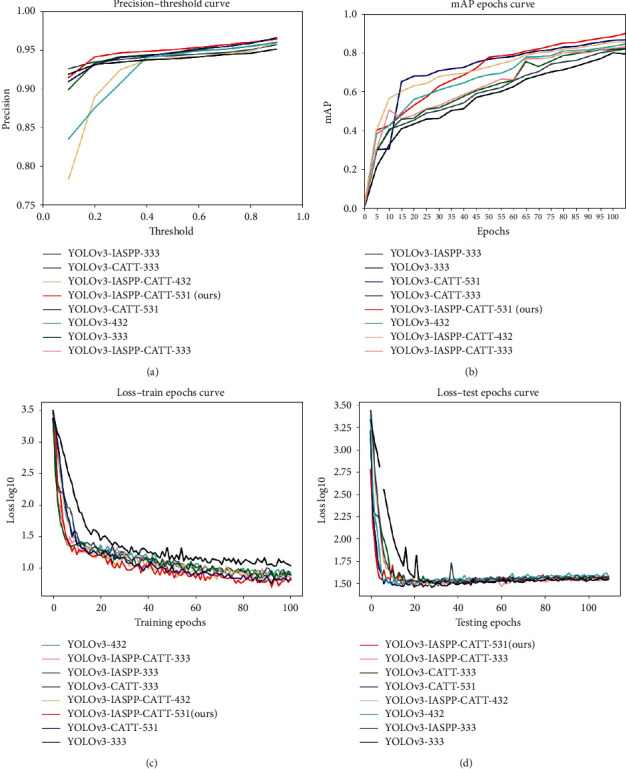
Evaluation curves of the eight algorithms. (a) Accuracy-Threshold curve. (b) mAP-Train epochs curve. (c) Loss-Train epochs curve. (d) Loss-Test epochs curve.

To comprehensively compare and analyze the advantages and disadvantages of the eight algorithms, we have drawn four curves in Figure 12 for comparative analysis. We use eight different colors to represent eight algorithms among the four curves. The color of the specific algorithm is shown in the four subpictures.

Figure 12(a) shows the relationship between the accuracy and threshold of object detection. It is known from (a) that as the threshold level increases, object recognition accuracy also increases. During detection, the accuracy and threshold provided by the red line model are significantly higher than the other three models, reflecting the superiority of the YOLOv3-IASPP-CATT-531 (ours) algorithm.

The mAP (mean average precision) provides a single-figure measure of quality across recall levels. Among evaluation measures of different object detection algorithms, mAP has been excellent at discrimination and stability. In the experiments of this paper, the training epochs have been iterated a total of 100. We have the mAP value as the *y*-axis, the range is from 0 to 100%, and the number of iterations as the *x*-axis, which ranges from 0 to 100. We can see from Figure 12(b) that when the number of training epochs reaches 100, the best performing red curve mAP got the maximum of 93.07%.

Figures 12(c) and 12(d) show the train-loss and test-loss value curve change with the iterations of the eight algorithms. It can be observed from the decreased degree of the loss of these two figures that although the eight algorithms can reach a stable value in the end regardless of training or testing, the original YOLOv3 algorithm continuously decreases slowly at first. The YOLOv3-IASPP-CATT-531 proposed in this paper always drops the fastest.

Through the above experimental results, the joint application of the anchor assignment rules based on the effective receptive field, the improved-ASPP, and the channel attention proposed in this paper has indeed achieved good results in the object detection of PCB assembly scenes.

#### 4.5.3. Comparisons of Detection Performance between Our Algorithms and Other Methods

To further illustrate the performance of the algorithm, the above eight algorithms of YOLOv3 are selected to compare with the current advanced anchor-based target detection methods, including Faster R-CNN [63], SSD [64], and YOLOv4 [15].

Here, we are concerned about the detection accuracy (mAP) and the computational complexity. Here, we use the number of parameters (use Mb as a unit) and FLOPs (floating point operations) to measure the complexity of the model. The former describes how many parameters are needed to define this complex network, i.e., the storage space required to store the model. The latter describes how much computation is required for data to pass through such a complex network, i.e., the calculation force needed to use the model. A low-complexity CNN has a small number of parameters and few FLOPs.  Table 8 shows the accuracy and complexity of eleven algorithms. SSD has the smallest parameters, YOLOV3-CATT-333 has the fewest computational complexity, and YOLOV3-IASPP-CATT- 531 (OURS) has the highest detection accuracy. In summary, YOLOV3-IASPP-CATT-531(OURS) is superior to other algorithms in the balance of high detection accuracy and low computational complexity.

### 4.6. Discussion

We have shown that by calculating the ERF size corresponding to each grid of the anchor allocation layer and using the ERF ranges of the three output layers to determine the allocation of anchors, the recognition accuracy and positioning accuracy of object detection in PCB assembly scenes can be improved. For small-size objects, we designed the improved-ASPP and application channel attention to expand the ERF. When these two modules perform feature extraction, more context information is added, and weight information is automatically assigned to different channels. They are sensitive and reliable. By visualizing the ERF of the grid at different positions of the three output layers, we have achieved the purpose of refined analysis inside the DCNN. By comparing the subjective detection images and objective statistical data of the eight algorithms in the test dataset, we found that the proposed method has shown superiority in object detection.

Although YOLOv3-IASPP-CATT-531 has obtained the advantage of high detection accuracy, there are still some potential limitations and challenges to improve its effectiveness further. First of all, within each ERF, there is a difference in the degree of activation. A strong degree of activation indicates that a strong possibility exists for the object, and a weak degree of activation indicates a poor possibility. When placing anchors, the current default is to place the center point of the anchor at the center point of each grid mapped back to the original image area. This point may be the weak activation area of the ERF. Hence, how to make the anchor and the strong activation area of ERF alignment is the next issue to be considered. Secondly, the number of anchors under the YOLOv3 model is nine by default. Although we use anchor allocation rules to change the number of each output layer adaptively, it does not reduce the density of anchors. For the training dataset, effectively reducing the number of anchors and increasing the detection speed while ensuring the detection effect is still challenging. Finally, after a refined analysis of the ERF, whether the smallest bounding rectangle of the active area of the ERF can be used in object orientation detection is still a mystery. Fortunately, according to recent experience, the current research status of deep feature alignment [65], dynamic learnable anchors [66], and object orientation detection [67] can all bring inspiration to solve the above problems. Therefore, future research related to anchors and ERF will continue to be in-depth in adapting to different object detection tasks, improving detection results, and increasing detection speed.

## 5. Conclusion

Electronic product manufacturing based on the background of intelligent manufacturing requires PCB assembly to be automated and intelligent. The visual object detection of the PCB assembly scene is the basis of the realization of intelligence. We try to solve the problem of object detection in the PCB assembly scene. Firstly, we proposed the PCB assembly scene object detection dataset, including three scenes before assembly, during, and after assembly. There are 21 classes of objects to be detected, mainly through-hole components and through-hole. Secondly, we deeply analyzed the ERF size corresponding to each grid on the three output layers of YOLOv3 and determined the ERF range of the three output layers. Again, we proposed an ERF-based anchor allocation rule using the anchor reallocated to optimize the classification and positioning of the bounding box. Finally, we propose an improved-ASPP and channel attention joint module for the small-size TH before and after the insertion, adding more context information to improve detection. Our proposed method has achieved the best results on object detection in PCB assembly scenes by experimental comparison.

## Figures and Tables

**Algorithm 1:**
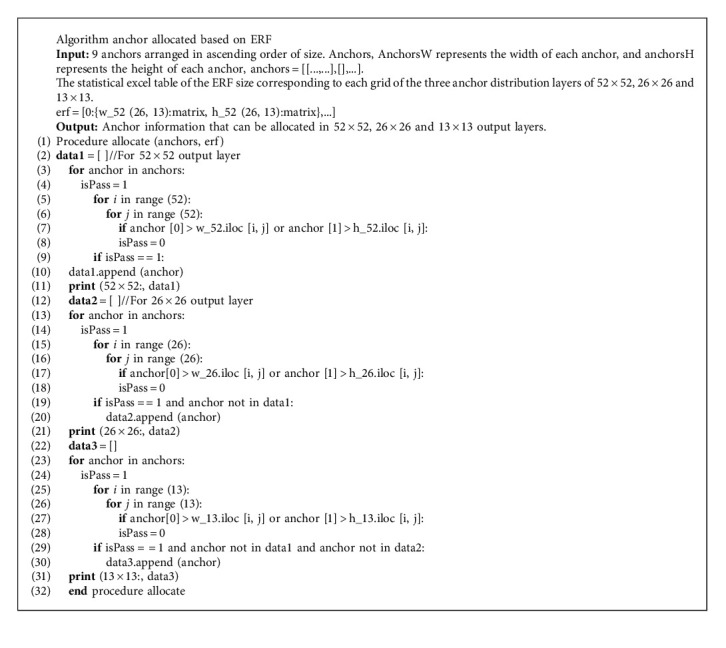
Anchor allocation algorithm.

**Table 1:** 13 × 13 ERF analysis.

Anchor allocation layer	Grid	ERF figure	ERF size (w × h)
13 × 13	1##1	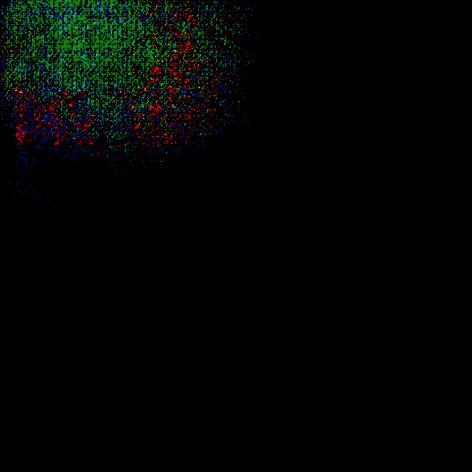 1##1 ERF figure	218 × 170
	13##1	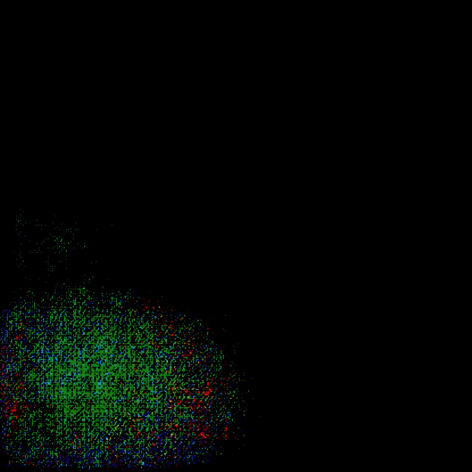 13##1 ERF figure	224 × 236
	7##7	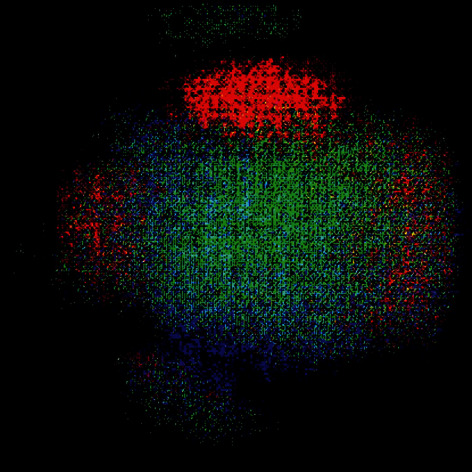 7##7 ERF figure	390 × 392
	8##12	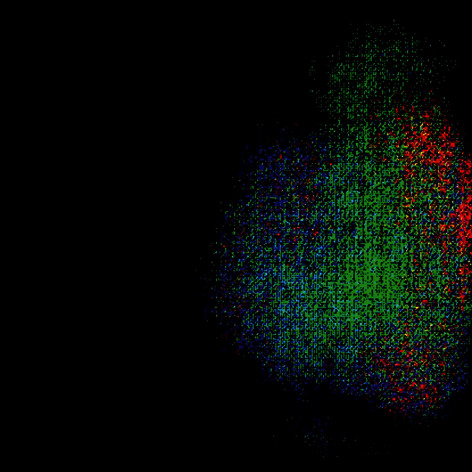 8##12 ERF figure	245 × 397
	13##13	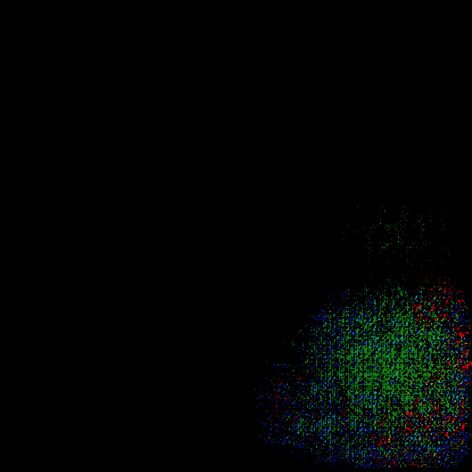 13##13 ERF figure	189 × 227

**Table 2:** 26 × 26 ERF analysis.

Anchor allocation layer	Grid	ERF figure	ERF size (w × h)
26 × 26	1##1	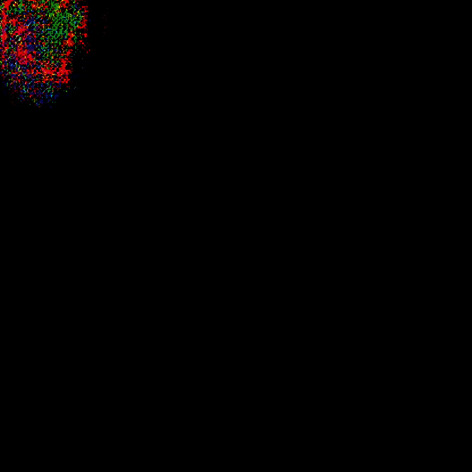 1##1 ERF figure	95 × 102
	8##6	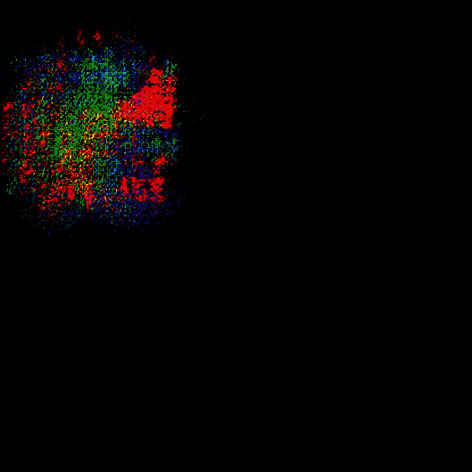 8##6 ERF figure	175 × 196
	13##13	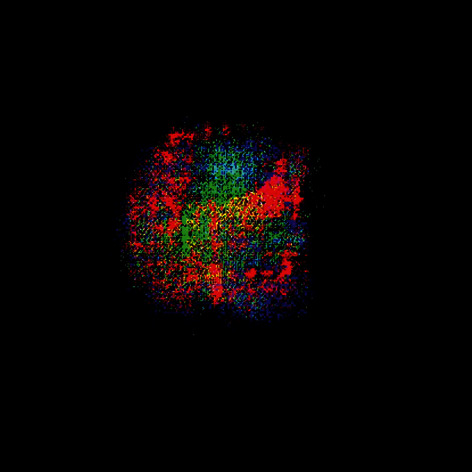 13##13 ERF figure	177 × 180
	19##22	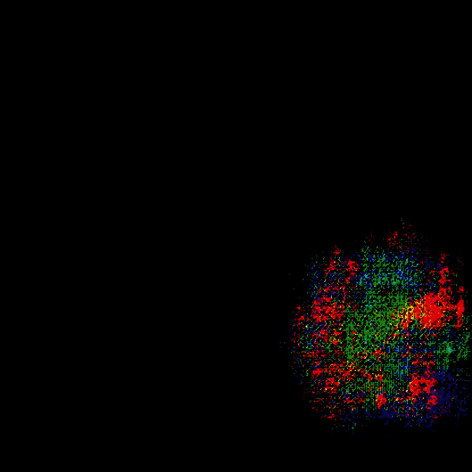 19##22 ERF figure	170 × 196
	26##26	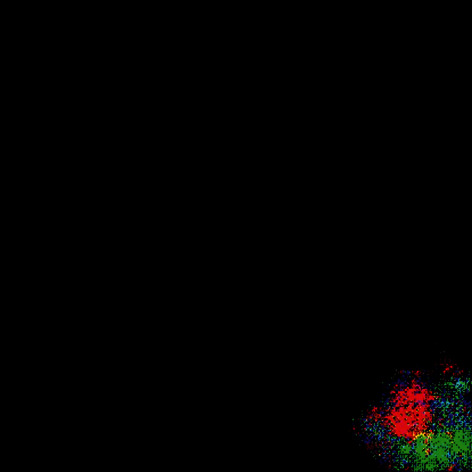 26##26 ERF figure	110 × 116

**Table 3:** 52 × 52 ERF analysis.

Anchor allocation layer	Grid	ERF figure	ERF size (w × h)
52 × 52	1##1	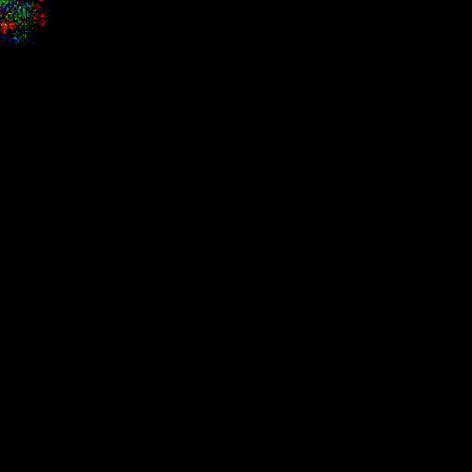 1##1 ERF figure	44 × 41
	3##22	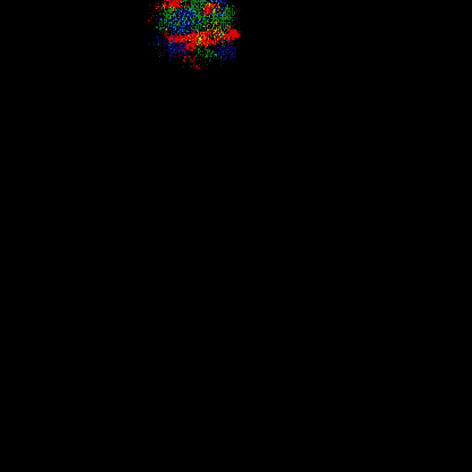 3##22 ERF figure	82 × 64
	27##27	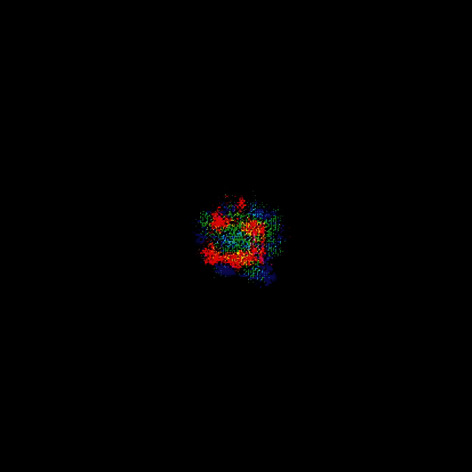 27##27 ERF figure	90 × 82
	45##9	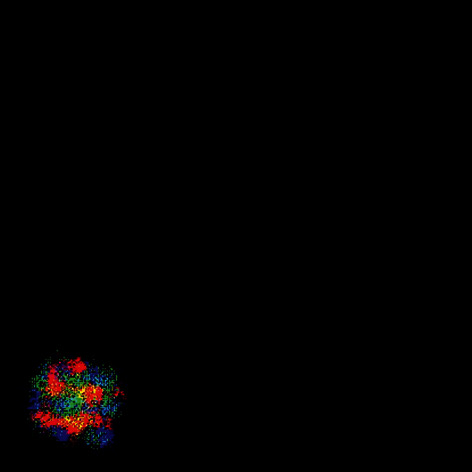 45##9 ERF figure	75 × 76
	52##50	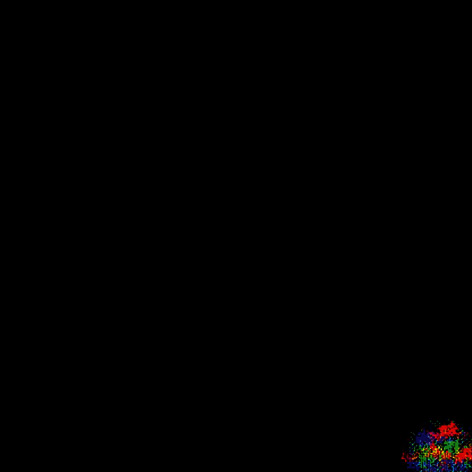 52##50 ERF figure	65 × 53

**Table 4:** Experimental parameter settings.

Parameter	Value
Train image size in pixels (height × width)	416 × 416
Number of categories	21
Training epochs	100
Train warmup epochs	10
Learn_rate_init	0.0001
Learn_rate_end	0.000001
Gradient descent	ADAM OPTIMIZER
Train batch size	12

**Table 5:** Algorithm name, improved content, and anchor allocation.

Algorithm name	Neck	52 × 52	26 × 26	13 × 13
YOLOv3-333	Original	[1, 3, 9, 14, 21, 22]	[22, 27, 30, 35, 41, 47]	[34, 47] [186, 12] [233, 15]
YOLOv3-IASPP-333	Improved ASPP	[1–3, 9, 14, 21, 22, 35]	[27, 30, 34, 41, 47, 47]	[186, 12] [233, 15]
YOLOv3-CATT-333	Channel attention			
YOLOv3-IASPP -CATT-333	Improved ASPP + channel attention joint module			
YOLOv3-432	Original			
YOLOv3-IASPP -CATT-432	Improved ASPP + channel attention joint module	[9, 14, 17, 21, 22, 22, 27, 32, 35, 41]	[30, 47] [34, 47] [186, 120]	[233, 151]
YOLOv3-CATT-531	Channel attention			
YOLOv3-IASPP -CATT-531 (ours)	Improved ASPP + channel attention joint module			

**Table 6:** AP for each class and mAP of eight algorithms.

Class	AP of eight algorithm							
	YOLOv3-333 (%)	YOLOv3-IASPP-333 (%)	YOLOv3-CATT-333 (%)	YOLOv3-IASPP-CATT-333 (%)	YOLOv3-432 (%)	YOLOv3-IASPP-CATT-432 (%)	YOLOv3-CATT-531 (%)	YOLOv3-IASPP-CATT-531 (ours)
3 (%) 0CTQ	69.83	70.80	74.11	75.19	74.58	80.06	86.06	92.58
AUIRFR	82.36	83.78	84.79	85.75	86.41	88.41	94.41	95.41
BUK7608	93.64	92.64	94.45	93.47	92.17	94.64	97.64	98.17
Cap100uF	80.00	86.67	81.22	87.15	87.49	85.29	85.29	87.49
Cap220uF	94.46	90.11	93.52	93.79	95.18	96.47	91.00	94.18
Cap22uF	90.24	90.49	92.19	90.22	90.74	90.38	90.38	92.74
Cap470uF	85.86	86.15	97.31	99.41	99.47	82.82	82.82	99.87
GK835	71.32	74.39	77.69	78.88	81.56	83.41	85.51	97.56
Inductance	88.68	87.27	89.12	92.78	91.84	90.80	90.80	91.84
Inserted100uF	93.62	80.65	94.63	95.52	96.45	89.13	89.13	87.45
Inserted220uF	89.01	90.10	90.23	90.23	88.56	89.01	89.01	88.56
Inserted22uF	84.72	86.87	88.42	86.35	87.27	88.13	87.13	90.27
Inserted470uF	67.27	74.12	74.49	71.25	69.46	74.31	80.89	89.46
InsertedInd	0.00	25.11	0.00	0.00	17.08	38.89	42.89	46.21
PCB	71.13	75.26	77.22	80.13	82.11	83.65	84.09	85.14
Pin100uF	61.90	76.19	77.19	67.19	74.11	77.41	77.54	84.21
Pin220uF	87.75	89.82	91.39	92.12	93.24	93.17	93.17	93.24
Pin22uF	87.50	88.15	79.12	83.21	88.16	91.67	90.67	92.16
Pin470uF	95.83	94.67	93.12	95.12	95.88	97.22	97.22	97.88
PinInd	80.20	87.04	91.09	93.25	91.14	86.79	86.79	91.14
SSG8	90.44	91.38	84.03	92.37	91.58	95.45	95.45	91.58
mAP	79.32	81.98	82.16	83.02	84.50	85.58	86.57	89.86

**Table 7:** The effectiveness of different components on mAP.

Row ID	Algorithm	Improved-ASPP	CATT	ERF-based anchor allocation	mAP@0.5
1	YOLOv3-333	✕	✕	✕	79.32%
2	YOLOv3-IASPP-333	√	✕	✕	81.98% + 2.66%
3	YOLOv3-CATT-333	✕	√	✕	82.16% + 2.84%
4	YOLOv3-IASPP-CATT-333	√	√	✕	83.02% + 3.70%
5	YOLOv3-432	✕	✕	√	84.50% + 5.18%
6	YOLOv3-IASPP-CATT-432	√	√	√	85.58% + 6.25%
7	YOLOv3-CATT-531	✕	√	√	86.57% + 7.24%
8	YOLOv3-IASPP-CATT-531 (ours)	√	√	√	89.86% + 10.54%

**Table 8:** Statistics of accuracy and complexity of eleven algorithms

Model	mAP (%)	Param (M)	FLOPs (G)
Faster R-CNN (Resnet50)	74.31	43.44	742.47
SSD (VGG16)	82.74	**28.52**	91.55
YOLOv4	88.86	64.05	90.74
YOLOv3-333	79.32	61.68	32.72
YOLOv3-IASPP-333	81.98	66.42	35.04
YOLOv3-CATT-333	82.16	58.92	**30.60**
YOLOv3-IASPP-CATT-333	83.02	69.93	35.35
YOLOv3-432	84.50	61,65	32.73
YOLOv3-IASPP-CATT-432	85.58	63,68	33.79
YOLOv3-CATT-531	86.57	58.89	30.61
YOLOv3-IASPP-CATT-531 (ours)	**89.86**	61.73	32.74

## Data Availability

The data used to support the findings of this study are available from the corresponding author upon request.
